# Supplementary material for: Nanotransistor-based gas sensing with record-high sensitivity enabled by electron trapping effect in nanoparticles
Source: Nat Commun. 2024 Jun 19;15:5259. doi: 10.1038/s41467-024-49658-3 (PMC11187184; doi:10.1038/s41467-024-49658-3)
Supplement: Supplementary file 1 — Supplementary Information [file 41467_2024_49658_MOESM1_ESM.pdf]

Supplementary Information

Nanotransistor-Based Gas Sensing with Record-High Sensitivity Enabled by Electron Trapping Effect in Nanoparticles

*Qitao Hu[1†], Paul Solomon[2], Lars Österlund[3], and Zhen Zhang[1]**

[1] Division of Solid-State Electronics, Department of Electrical Engineering, Uppsala University, BOX 65, SE-75121, Uppsala, Sweden

[2] IBM T. J. Watson Research Center, Yorktown Heights, NY 10598 USA

[3] Division of Solid-State Physics, Department of Materials Science and Engineering, Uppsala University, BOX 35, SE-75103, Uppsala, Sweden

[†]Current address: Department of Radiology, Stanford University, Stanford, CA 94305 USA

*Corresponding author: Prof. Z. Zhang, zhen.zhang@angstrom.uu.se

Supplementary Section 1: Device fabrication process flow

A CMOS-compatible top-down process was used to fabricate the SiNW-NAG FET device. The fabrication process is presented step by step in Supplementary Fig. 1.

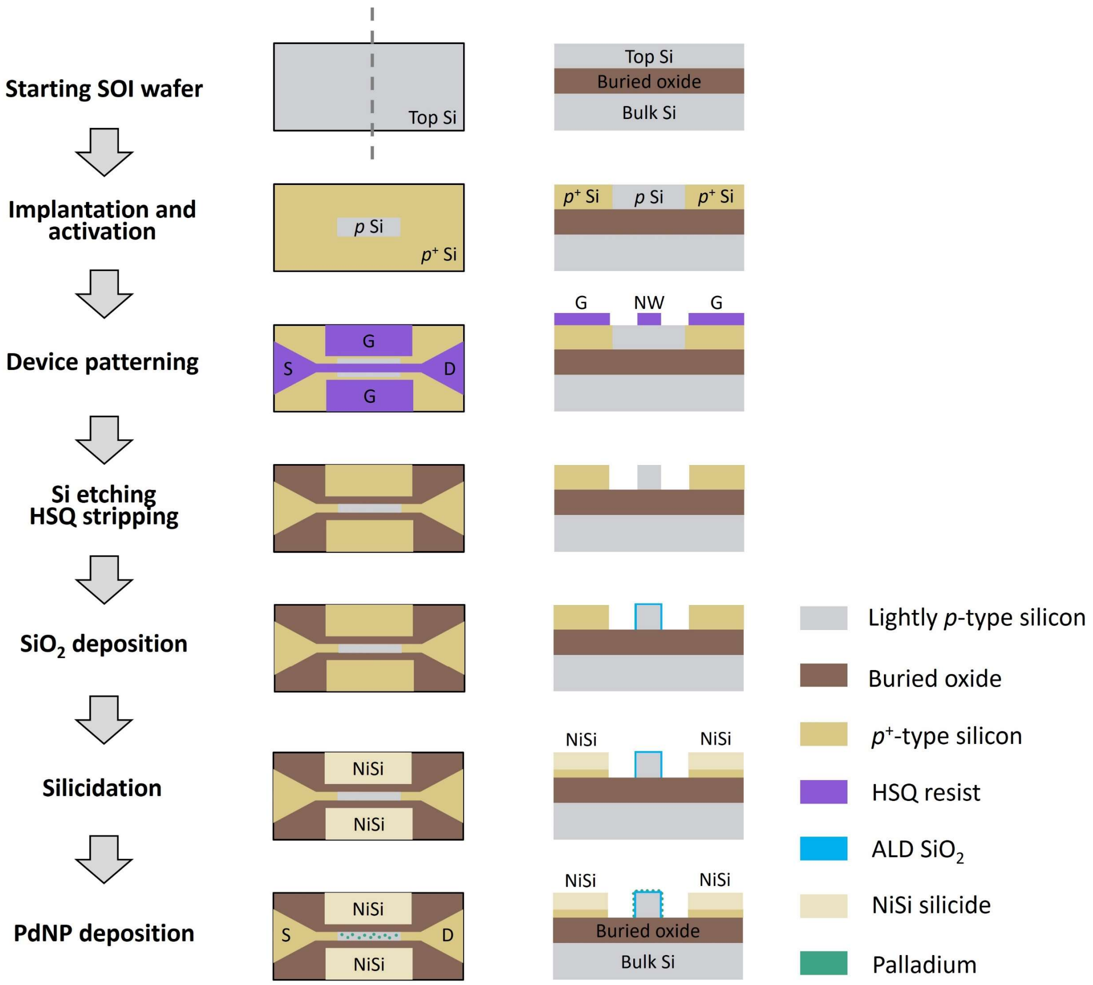

Supplementary Fig. 1 The top-view and cross-sectional schematic illustrations of the device at each step are presented. The dashed line in the top-view schematic indicates the position of cross-sectional cut.

Supplementary Section 2: Top-view SEM image of SiNW-NAG FET device

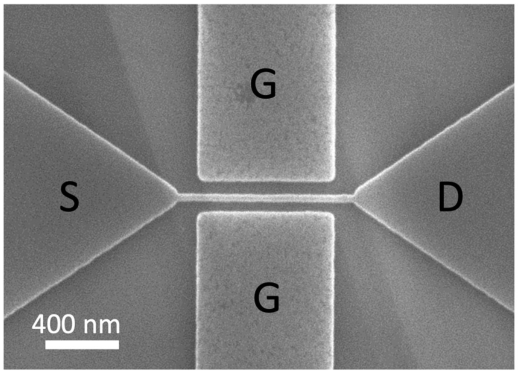

Supplementary Fig. 2 The top-view SEM image of the SiNW-NAG FET device, which indicates the 35 nm wide SiNW and the 50 nm wide air gaps between the side-gates and SiNW.

Supplementary Section 3: TCAD simulation of potential distribution

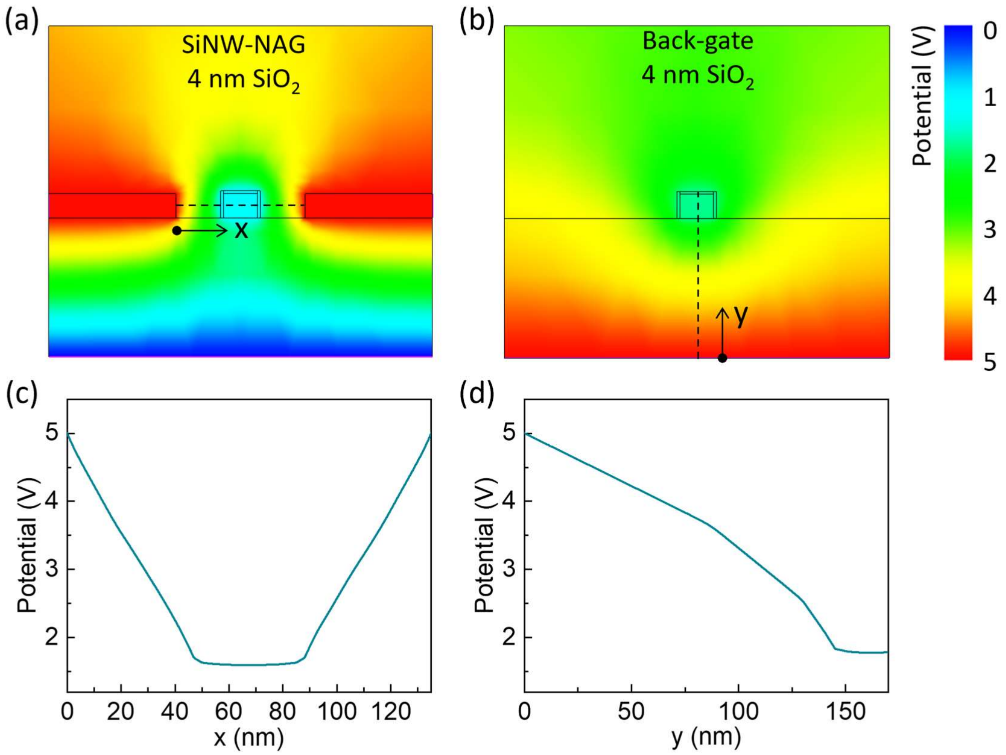

Supplementary Fig. 3 TCAD simulated cross-sectional profile of electrical potential at the SiNW center of (a) SiNW-NAG and (b) back-gate SiNW FET devices. (c) and (d) show the electrical potential variations along the dashed cutline in (a) and (b), respectively. V_G = 5 V and V_D = 1 V. In the SiNW-NAG FET, the voltage applied on the side-gates drops to the SiNW channel through the air gap. Thereby, the channel is coupled to the side-gate through the capacitance of air gap. In the back-gate SiNW FET, the gate voltage applied on the back-gate drops through the buried oxide, which means that the channel-gate coupling is realized through the capacitance of buried oxide.

Supplementary Section 4: *IV* characteristics

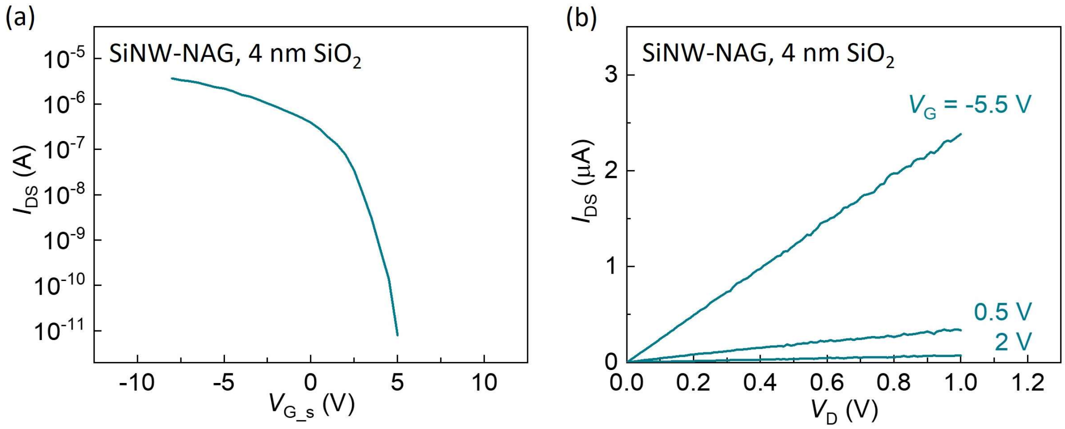

Supplementary Fig. 4 (a) Transfer and (b) output characteristics of the SiNW-NAG FET with 4 nm thick SiO_2 passivation layer were measured in air at room-temperature. The drain voltage was biased at 1 V in the transfer curve measurement. The voltage applied on the side-gates can effectively modulate I_{DS} in a broaden range with the on/off ratio over 10^6, which enables the gas sensing in a wide concentration range. The output curves suggest a good Ohmic contact performance.

Supplementary Section 5: Analysis of back-gate and SiNW-NAG FET devices

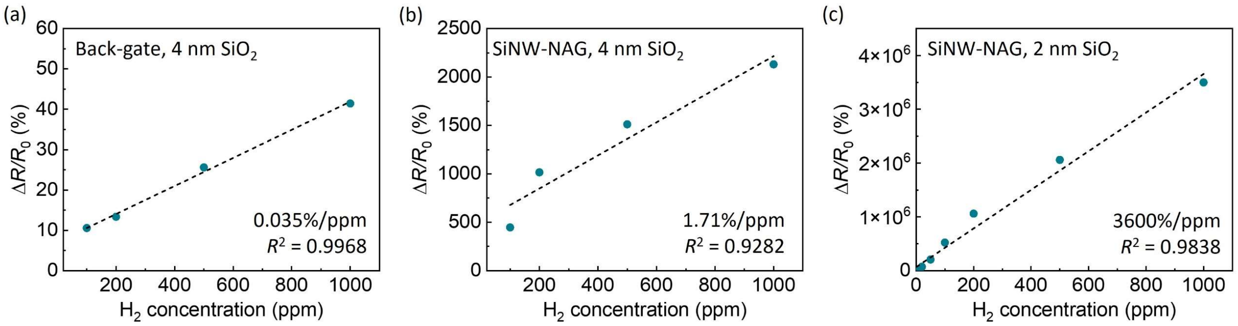

Supplementary Fig. 5 Responses to H_2 ($\Delta R/R_0$) of (a) 4 nm SiO_2 layer passivated back-gate, (b) 4 nm SiO_2 layer passivated SiNW-NAG, and (c) 2 nm SiO_2 layer passivated SiNW-NAG FET devices. H_2 sensitivity of 0.035%/ppm (back-gate, 4 nm SiO_2), 1.71%/ppm (SiNW-NAG, 4 nm SiO_2), and 3600%/ppm (SiNW-NAG, 2 nm SiO_2) is extracted.

Based on the International Union of Pure and Applied Chemistry (IUPAC) definition, the minimum detectable signal is three times larger than the baseline noise (σ). Therefore, the lower limit of detection (LOD) of our H_2 sensors can be calculated using

$$\mathrm{LOD} = 3\frac{\sigma}{I_{\mathrm{DS0}}}/\mathrm{sensitivity},$$

where I_{DS0} is the baseline current and I_{DS0} = 300 nA. The baseline noise σ is obtained as the standard deviation of the stabilized baseline current data before exposure to any H_2. The sensitivity, σ, and LOD of back-gate and SiNW-NAG FET devices are summarized in Table S1.

Table S1 Detection limit analysis of back-gate and SiNW-NAG FET devices.

Device	Sensitivity	σ	LOD
Back-gate, 4 nm SiO_2	0.035%/ppm	1.7×10^{-9} A	48 ppm
SiNW-NAG, 4 nm SiO_2	1.71%/ppm	4.6×10^{-9} A	2.7 ppm
SiNW-NAG, 2 nm SiO_2	3600%/ppm	1.6×10^{-8} A	4.4 ppb

Supplementary Section 6: TCAD simulation of carrier profile

Supplementary Fig. 6 presents the TCAD simulation results of the cross-sectional profile of hole density at the center of the SiNW. The SiO_2 passivation layer is 4 nm thick, which suggests that the primary H_2 sensing mechanism is the capacitive coupling of the PdNP work function change. The H_2-induced PdNP work function change is modeled in TCAD environment by setting a dipole layer (50 mV potential drop in the simulation) at the interface between SiO_2 passivation layer and surrounding air region[1, 2]. The hole densities along the dashed cutlines in the cross-sectional profiles with H_2 off/on are also plotted in Supplementary Fig. 6 (b). The TCAD simulation results show that the modulation of carrier density in the main channel with the presence of H_2 is much more significant in the SiNW-NAG FET device than in the conventional back-gate FET device.

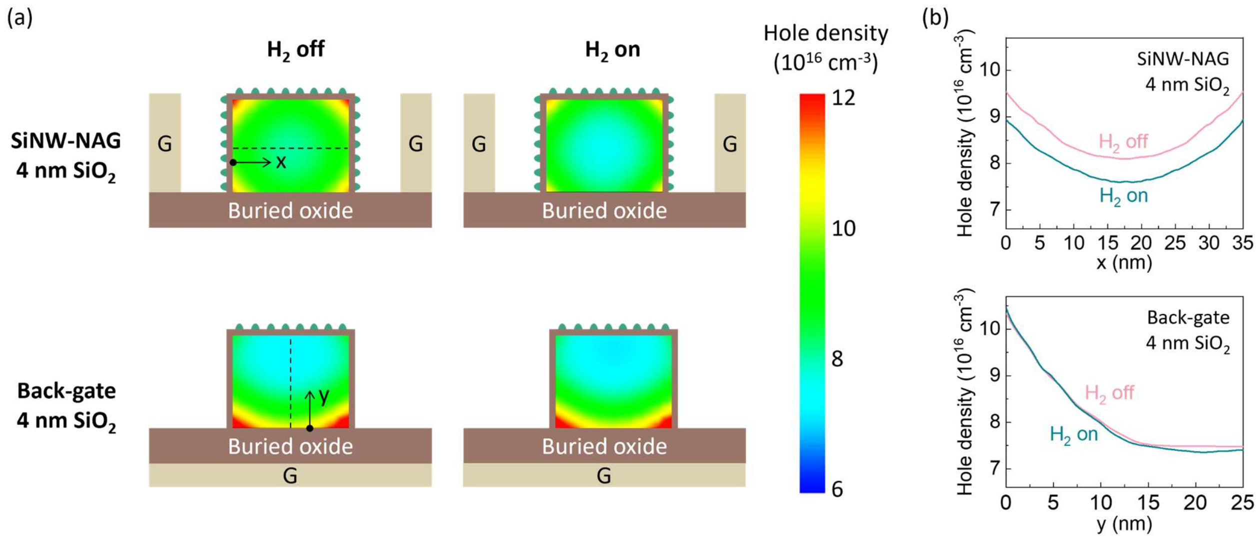

Supplementary Fig. 6 (a) TCAD simulation of the cross-sectional distribution of hole at the SiNW center of the 4 nm SiO_2 layer passivated SiNW-NAG FET and conventional back-gate SiNW FET devices pre and post H_2 exposure. (b) Hole density along the dashed cutlines in (a) pre and post H_2 exposure. V_D = 1 V and initial I_D = 300 nA.

Supplementary Section 7: Device noise of SiNW-NAG FET

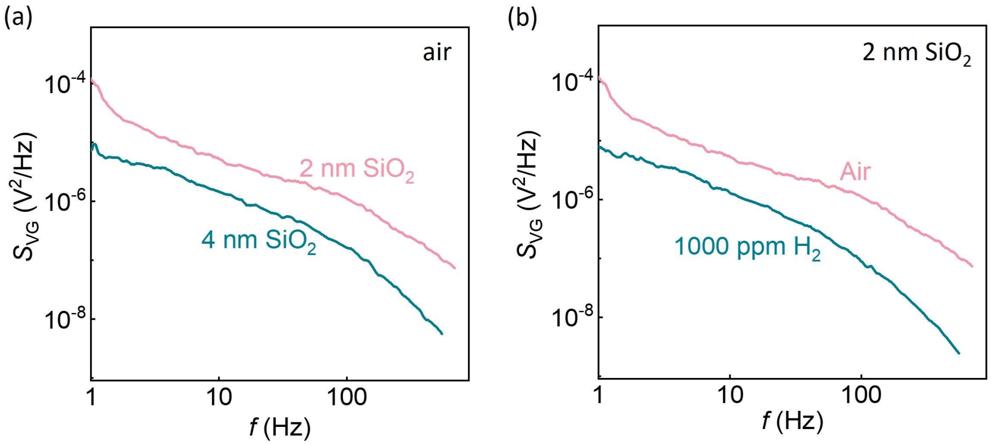

Supplementary Fig. 7 Power spectrum density of gate voltage referred noise S_{VG} of the SiNW-NAG FET devices (a) with 2 and 4 nm thick SiO_2 passivation layer measured in air and (b) with 2 nm thick SiO_2 passivation layer measured in air and 1000 ppm H_2.

Supplementary Section 8: Equivalent circuit of SiNW-NAG FET

The complete equivalent circuit of SiNW-NAG FET device with 2 nm SiO_2 is plotted in Supplementary Fig. 8. C_{NAG} and C_{SiO_2} are geometric capacitances of air gap and SiO_2 passivation layer, respectively. C_{Si} is the capacitance of the SiNW channel and C_{PdNP} is the capacitance associated with the electron trapping/detrapping processes with the PdNPs. C_{Si} and C_{PdNP} are connected to the source (V_S) because they are in equilibrium with the source Fermi level. The resistor R_{Tun} is the tunneling resistance across the SiO_2 passivation layer. Considering the thickness of the SiO_2 layer, C_{SiO_2} is significantly larger than C_{Si}, so C_{SiO_2} can be neglected and the equivalent circuit in Fig. 2c is obtained. For the thick SiO_2 layer, R_{Tun} becomes very large, cutting off the branch of C_{PdNP}. Consequently, the equivalent circuit in Fig. 1c (right) is derived.

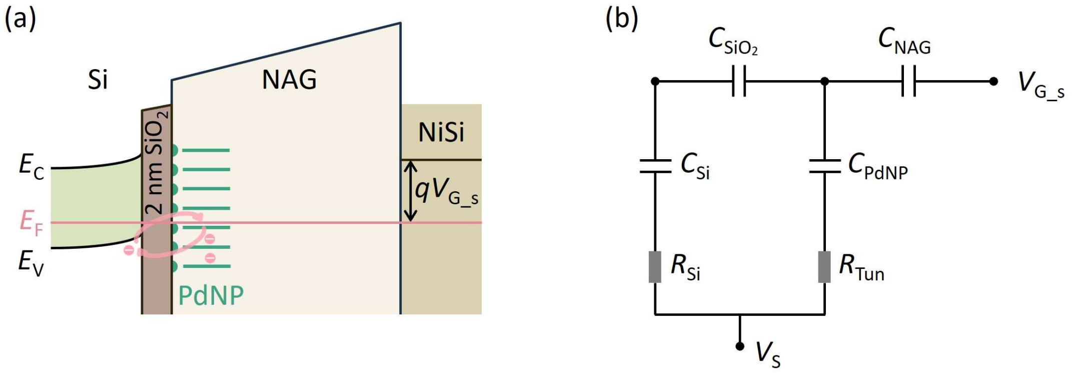

Supplementary Fig. 8 (a) Schematics of the energy diagram and (b) equivalent circuit of 2 nm thick SiO_2 layer passivated SiNW-NAG FET device.

Supplementary Section 9: Capacitance Analysis

The energy diagrams and equivalent circuits of the SiNW-NAG FET devices without and with PdNPs are exhibited in Supplementary Fig. 9a and b, respectively. In the device with bare SiO_2 surface, the capacitances of Si channel (C_{Si}) and air gap (C_{NAG}) are connected in series (see Supplementary Fig. 9a). The subthreshold slope without PdNPs ($SS_{no\text{-}Pd}$) is

$$SS_{no-} \quad = \left(1 + \frac{C_{Si}}{C_{NAG}}\right) \times 60\text{mV/dec.}$$

Considering $SS_{no\text{-}Pd} = 0.34$ V/dec extracted from Fig. 2b in the I_{DS} range from 10^{-10} to 10^{-8} A, $C_{Si} = 4.7C_{NAG}$ can be estimated.

When the device is coated with PdNPs, the PdNPs contribute an equivalent capacitance (C_{PdNP}) connected to C_{Si} in parallel. The subthreshold slope with PdNPs (SS_{Pd}) is

$$SS_{Pd} = \left(1 + \frac{C_{Si} + C_{PdNP}}{C_{NAG}}\right) \times 60\text{mV/dec.}$$

Since $SS_{Pd} = 5.98$ V/dec is extracted from Fig. 2b in the I_{DS} range from 10^{-10} to 10^{-8} A, $C_{Si} + C_{PdNP} = 98.7C_{NAG}$ can be estimated. Considering the same device geometry, it is reasonable to assume that both C_{Si} and C_{NAG} with the PdNPs are closing to them with the bare surface. Consequently, we can estimate that $C_{PdNP} = 94.0C_{NAG} = 20.0C_{Si}$, which indicates that C_{PdNP} is significantly larger than C_{Si}.

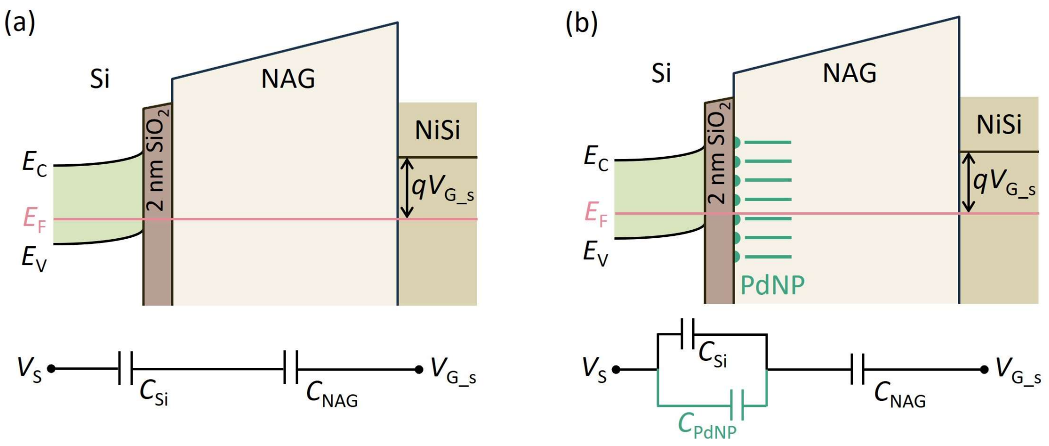

Supplementary Fig. 9 Schematics of the energy diagram and equivalent circuits of 2 nm thick SiO_2 layer passivated SiNW-NAG FET devices (a) without and (b) with the PdNP coating.

Supplementary Section 10: Sensing reproducibility test

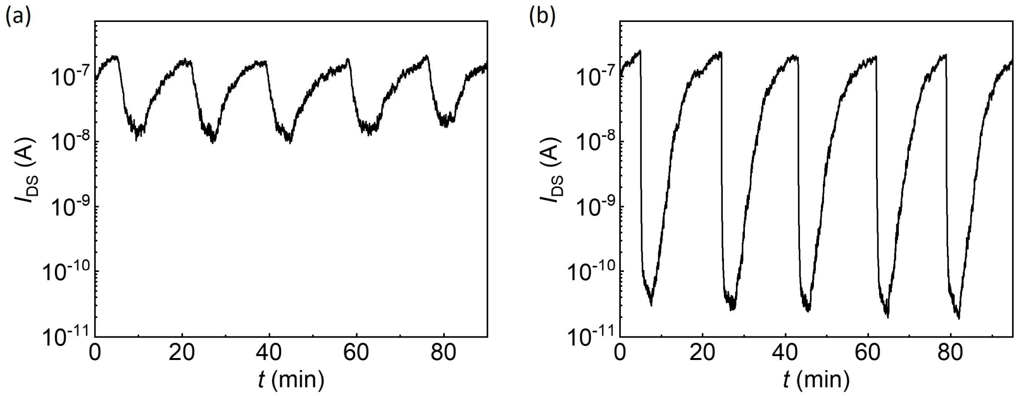

Supplementary Fig. 10 The sensing reproducibility of the SiNW-NAG FET device with 2 nm SiO_2 layer is demonstrated at (a) 2 and (b) 100 ppm H_2.

Supplementary Section 11: Analysis of variation of Fermi level in SiNW

The energy diagram and effective circuit is shown in Supplementary Fig. 11. To simply the analysis, all capacitances are assumed as constant. Once the energy of the PdNPs (E_{PdNP}) is modulated with H_2, the charge trapped the PdNPs is changed accordingly:

$$\Delta Q = C_{\mathrm{PdNP}} \Delta E_{\mathrm{PdNP}}.$$

ΔQ is in non-equilibrium state; ΔQ is shared with the capacitances of C_{Si}, C_{PdNP} and C_{NAG} to reach a new equilibrium. The charge shared with C_{PdNP} at equilibrium state is

$$\Delta Q_{\mathrm{PdNP}} = \Delta Q \frac{C_{\mathrm{PdNP}}}{C_{\mathrm{Si}} + C_{\mathrm{PdNP}} + C_{\mathrm{NAG}}}.$$

Since C_{Si} connected to C_{PdNP} in parallel, the voltage variation of C_{Si} at equilibrium state, *i.e.*, Fermi level variation ΔE_{F}, equals that of C_{PdNP}:

$$\Delta E_{\mathrm{F}} = \frac{\Delta Q_{\mathrm{PdNP}}}{C_{\mathrm{PdNP}}} = \frac{C_{\mathrm{PdNP}}}{C_{\mathrm{Si}} + C_{\mathrm{PdNP}} + C_{\mathrm{NAG}}} \Delta E_{\mathrm{PdNP}}.$$

Considering $C_{\mathrm{PdNP}} >> C_{\mathrm{Si}} >> C_{\mathrm{NAG}}$, ΔE_{F} is approaching ΔE_{PdNP}. Take the analysis results in Supplementary Section 9, $C_{\mathrm{PdNP}} = 94.0 C_{\mathrm{NAG}}$ and $C_{\mathrm{Si}} = 4.7 C_{\mathrm{NAG}}$, we can estimate $\Delta E_{\mathrm{F}} = 94.3\%\ \Delta E_{\mathrm{PdNP}}$. Such analysis indicates the Fermi level in the SiNW channel closely follows the energy of the PdNPs.

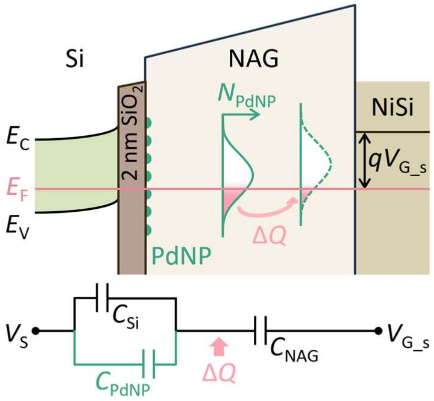

Supplementary Fig. 11 Schematics of the energy diagram and equivalent circuits of 2 nm SiO_2 passivated SiNW-NAG FET device with the PdNPs coating.

Supplementary References

1. Tov, I. S. et al. Hydrogen induced dipole layer in Pd-SiO_2 based gas sensors. *2022 IEEE Sensors Applications Symposium (SAS)*, Sundsvall, Sweden, 1–6 (2022).

2. Varghese, A., Eblabla, A. & Elgaid, K. Modeling and simulation of ultrahigh sensitive AlGaN/AlN/GaN HEMT-based hydrogen gas detector with low detection limit. *IEEE Sens. J.* **21**, 15361–15368 (2021).
